# Characterisation of cuticular inflation development and ultrastructure in *Trichuris muris* using correlative X-ray computed tomography and electron microscopy

**DOI:** 10.1038/s41598-020-61916-0

**Published:** 2020-04-03

**Authors:** James D. B. O’Sullivan, Sheena M. Cruickshank, Tobias Starborg, Philip J. Withers, Kathryn J. Else

**Affiliations:** 1 Henry Royce Institute, Department of Materials, The University of Manchester, Oxford Road, Manchester, M13 9PL United Kingdom; 2 The Lydia Becker Institute of Immunology and Inflammation, The University of Manchester, Oxford Road, Manchester, M13 9PL United Kingdom; 3 Wellcome Centre for Cell Matrix Research, The University of Manchester, Oxford Road, Manchester, M13 9PL United Kingdom

**Keywords:** Microbiology, Parasitology, Imaging techniques

## Abstract

The parasitic nematode *Trichuris trichiura* is a significant burden on public health in developing countries, and currently available drugs exhibit a poor cure rate. Worms live within a specialised tunnel of host intestinal epithelial cells and have anterior-ventral projections of the cuticle termed “cuticular inflations”, which are thought to be involved in host-parasite interactions. This work aimed to characterise structure and suggest a function of cuticular inflations in the most tractable and widely-used model of trichuriasis, *Trichuris muris*. Using scanning electron microscopy, we show for the first time that most cuticular inflations develop between the second and third larval moults. Correlative X-ray computed tomography (CT)-steered Serial Block Face Scanning Electron Microscopy (SBF-SEM) and transmission electron microscopy enabled ultrastructural imaging of cuticular inflations, and showed the presence of an additional, web-like layer of cuticle between the median and cortical layers of the inflation. Additionally, we characterised variation in inflation morphology, resolving debate as to the inflations’ true shape *in situ*. Cells underlying the inflations had many mitochondria, and we highlight their potential capacity for active transport as an area for future investigation. Overall, insights from the powerful imaging techniques used provide an excellent basis for future study of cuticular inflation function.

## Introduction

Trichuriasis, caused by the parasitic nematode *Trichuris trichiura*, is estimated to infect almost half a billion people worldwide^1^. Infection is propagated via the faecal-oral route. Larval worms hatch from the eggs in the caecum and penetrate the epithelial cell layer of the mucosa. The larvae generate a tunnel of dead host epithelial cells^2^ which lengthens to accommodate the developing worm, as it moults four times to reach adulthood^3^. During adulthood, the anterior two-thirds of the worm remains embedded within the tunnel, whilst the posterior hangs free in the lumen in order to facilitate mating and egg-laying^3^. *Trichuris* infection causes detrimental pathology when worm burdens are high, including colitis-like symptoms, anaemia, rectal prolapse and impaired cognitive development^4–6^. Disappointingly, current control efforts are running behind the established targets for reducing prevalence^7^, partly due to poor cure rate of existing anthelminthic drugs^8^. Therefore, there is need to develop more efficacious drugs for the fight against trichuriasis^9^.

One method of identifying novel drug targets is to increase our understanding of the host-parasite interactions at the attachment site, for instance by clarifying the mechanisms behind immunomodulation by the worm^10^. Very little is currently known about the mechanisms of tunnel formation and maintenance^11^, and a greater understanding of *Trichuris* anatomy and physiology represents an unexploited opportunity to develop new therapies. Within the tunnel, the worm anterior exhibits two notable sets of surface structures thought to be involved in maintaining the niche; firstly the bacillary cells, which lie underneath small bacillary pores covering the ventral surface of the worm in a region termed the bacillary band, and secondly the cuticular inflations. Cuticular inflations are projections of the cuticle of 10–20 µm diameter in the bacillary band, reported as either a cup-like or bulbous in appearance, and are currently of unknown function. Understanding the morphology and composition of surface structures such as the cuticular inflations is a necessary first step in determining their function, and future avenues for potential novel therapies.

Electron microscopy has been an important tool in determining bacillary cell function, because it enabled the identification of a highly folded apical membrane, suggesting absorptive function^12^. Subsequently, more hypothesis-driven approaches utilising fluorescence microscopy have given more evidence of a role in glucose and small molecule uptake^13,14^. The cuticular inflations have been remarked upon in investigations of *Trichuris* classification^15^. However, with only one ultrastructural study existing in the dog whipworm *Trichuris vulpis*^16^, composition of the inflations remains poorly understood. Even the true exterior morphology of cuticular inflations is still uncertain, as in Scanning Electron Microscopy (SEM) images two forms are presented: either convex or concave. This differential morphology is currently attributed to the drying process (freeze-drying or critical point drying) occurring during sample preparation causing naturally hydrated convex inflations to collapse into the concave form^16^. Since the late 20^th^ century, the mouse whipworm *Trichuris muris* has emerged as the most useful and tractable model of trichuriasis^11,17^. *T. muris* is the most suitable model for future hypothesis-driven investigation into cuticular inflations because larval development is well-characterised^3^, allowing detailed study of worm development. Additionally, a richer understanding of the epithelial niche and host-parasite interactions has been generated in the mouse model, allowing new ultrastructural information from *Trichuris* to be added to an extensive existing mosaic of immunological and cytological knowledge.

Cuticular inflations are challenging to image by electron microscopy. They are difficult to see with the naked eye and are far more sparsely placed along the bacillary band than bacillary cells. Therefore, inflations are hard to find by sectioning the resin block blindly. Furthermore, inflations must be sectioned longitudinally in order to view their internal structure in addition to the underlying tissue, meaning that correct orientation of tissue is also necessary. Here we present correlative tomography as an approach which addresses these issues and allows for efficient imaging of multiple inflations within the same worm. X-ray computed tomography (CT) was used to non-destructively generate a 3D image of the interior sample microstructure, which allowed the steering of subsequent milling and sectioning to a desired region of interest for further electron microscopy^18–20^. Combining this approach with Serial Blockface Scanning Electron Microscopy (SBF-SEM), in which the sample is serially sectioned and imaged, allowed the acquisition of 3D datasets over multiple regions of interest in one sample. Furthermore Transmission Electron Microscopy (TEM) was employed in a similar targeted manner to provide ultrastructural insights at even higher resolution. Utilising this approach, we are able to characterise the structure and composition of cuticular inflations in *Trichuris muris*, and identify for the first time their development in the larval stages of the parasite.

## Results

### SEM examination of the development of cuticular inflation

**Figure 1:**
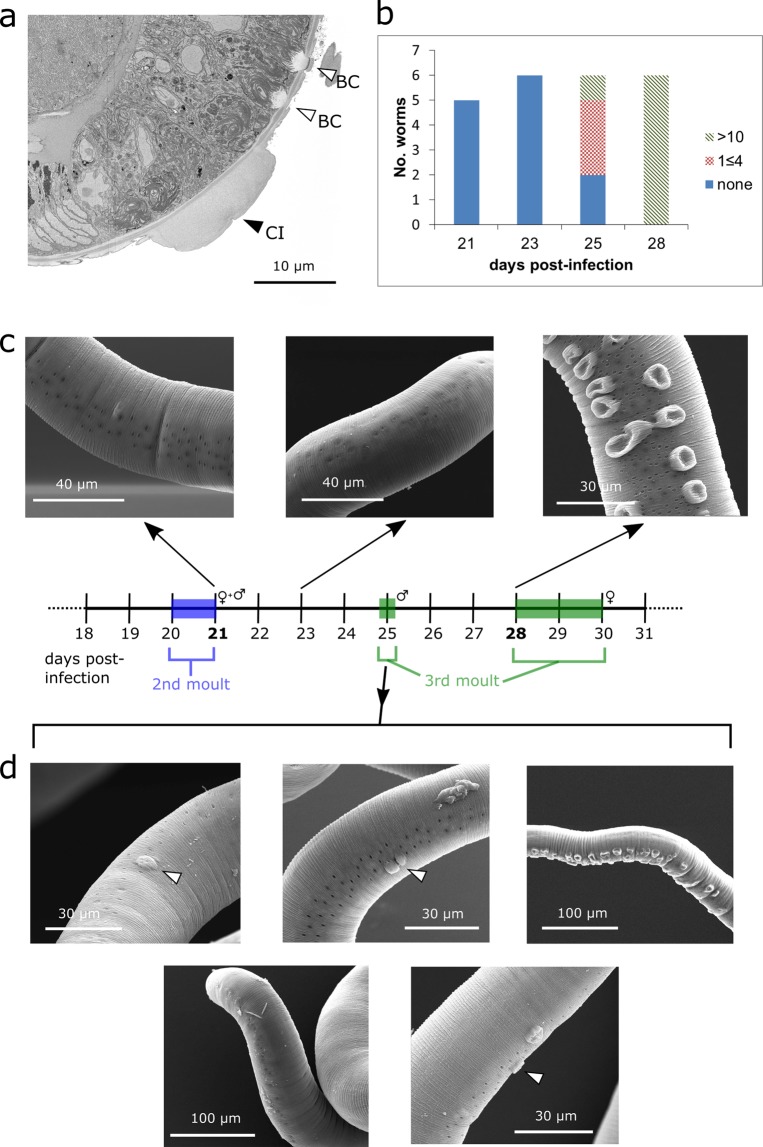
A developmental time-course of cuticular inflations generated using SEM. (**a**) Backscattered electron SEM cross section of adult *T. muris* after heavy metal staining and resin-embedding, showing the surface-projecting cuticular inflation (CI), as well as the sub-surface bacillary cells (BC). (**b**) Histogram of SEM data showing frequency of worms exhibiting no inflations, between 1 and 4 inflations, and more than 10 inflations at time-points during development. (**c**) Example SEM images from worms 21, 23, 25 and 28 days PI. No inflations can be seen at day 21 and 23 PI. At day 28 post-infection, all worms exhibited more than 10 cuticular inflations. (**d**) At 25 days PI three worms showed one or two inflations (◀), in contrast with one worm which had none and another which exhibited many inflations.

Cuticular inflations are outgrowths of the cuticle, which are separate from the bacillary cells that line much of the bacillary band as shown by Backscattered Electron Microscopy (BSEM) of a stained and resin-embedded sample (Fig. 1a). Standard SEM was used to image female worms that had been extracted from the host gut at days 21, 23, 25, and 28 post-infection (PI) in order to track the morphological development of the cuticular inflations (Fig. 1b). At day 21 and day 23 post-infection, none of the worms imaged (n = 5, 6) exhibited fully formed cuticular inflations (Fig. 1c). Day 25 PI was the first time-point at which worms definitively began to exhibit cuticular inflations (Fig. 1d). In three of these worms, either one or two convex cuticular inflations were present. One of the 25 day-old worms analysed exhibited over 10 cuticular inflations along the anterior bacillary band. However, two 25 day PI worms exhibited no evidence of cuticular inflations. In contrast, at day 28, all the worms imaged exhibited more than 10 cuticular inflations (Fig. 1c). A developmental window was therefore determined, in which the majority of visible cuticular inflation development occurred in worms between days 25 and 28 post-infection, between the second and third moults, although inflation development was not always synchronous between worms.

### SBF-SEM of cuticular inflation ultrastructure

**Figure 2:**
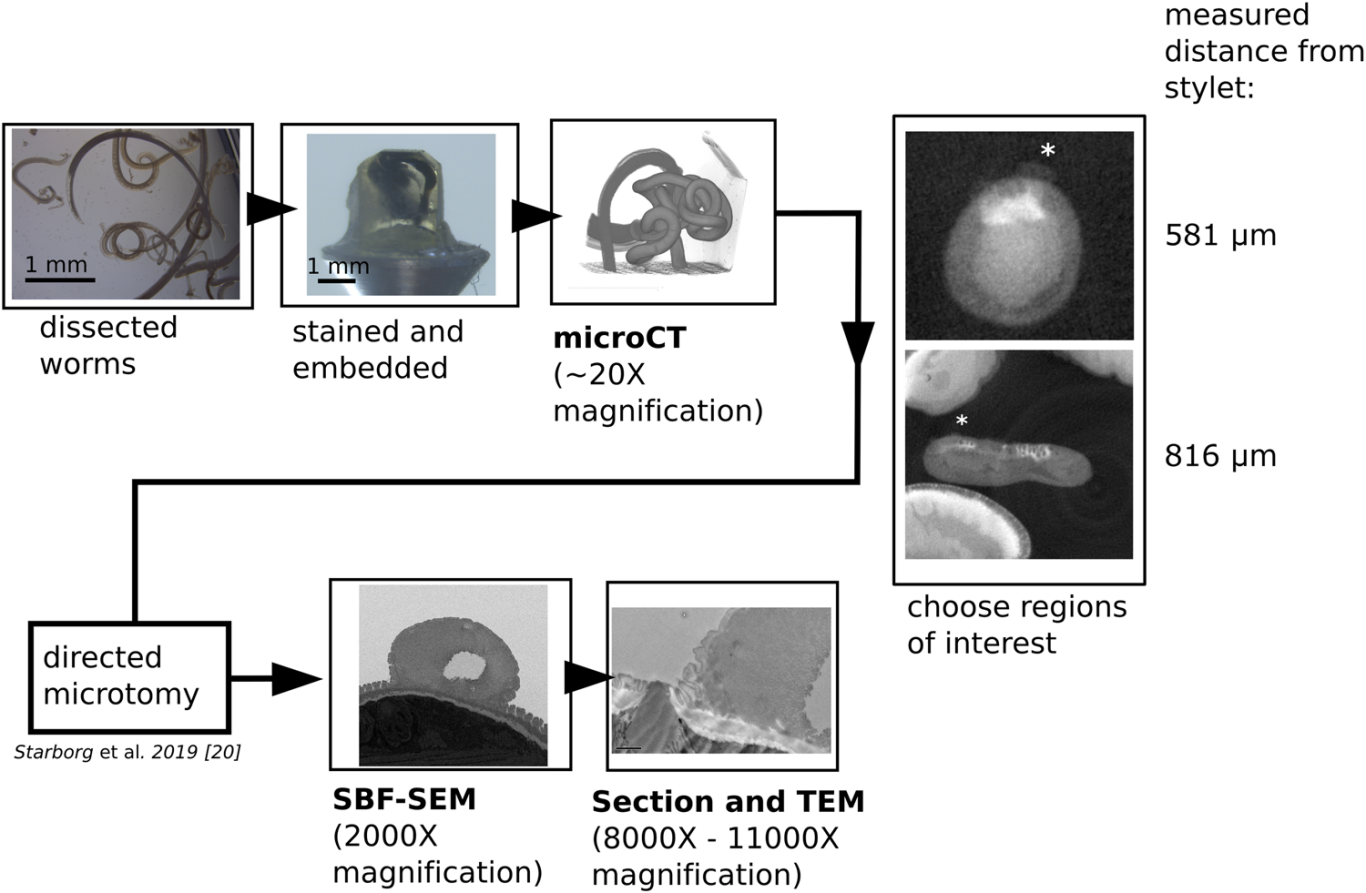
Schematic of the correlative workflow facilitating targeted electron microscopy of cuticular inflations. The worm is dissected from the host 35 days after initial infection, and processed for SBF-SEM. The worm is stained with osmium tetroxide, uranyl acetate and lead aspartate, and is embedded in epoxy resin. CT provides a virtual “map” of the tissue from which regions of interest, including the cuticular inflations (*) can be identified. After trimming the sample to expose the desired region of interest, SBF-SEM and TEM may be carried out.

A correlative tomography workflow incorporating X-ray CT was used to steer the SBF-SEM of stained and resin embedded worms. In this approach^20^, the CT images were used to select the regions for SBF sectioning, and the sample was accordingly trimmed such that multiple cuticular inflations at different regions of the bacillary band were captured and imaged in 3D by SEM. In total, two worms were imaged using this protocol (Fig. 2).

**Figure 3:**
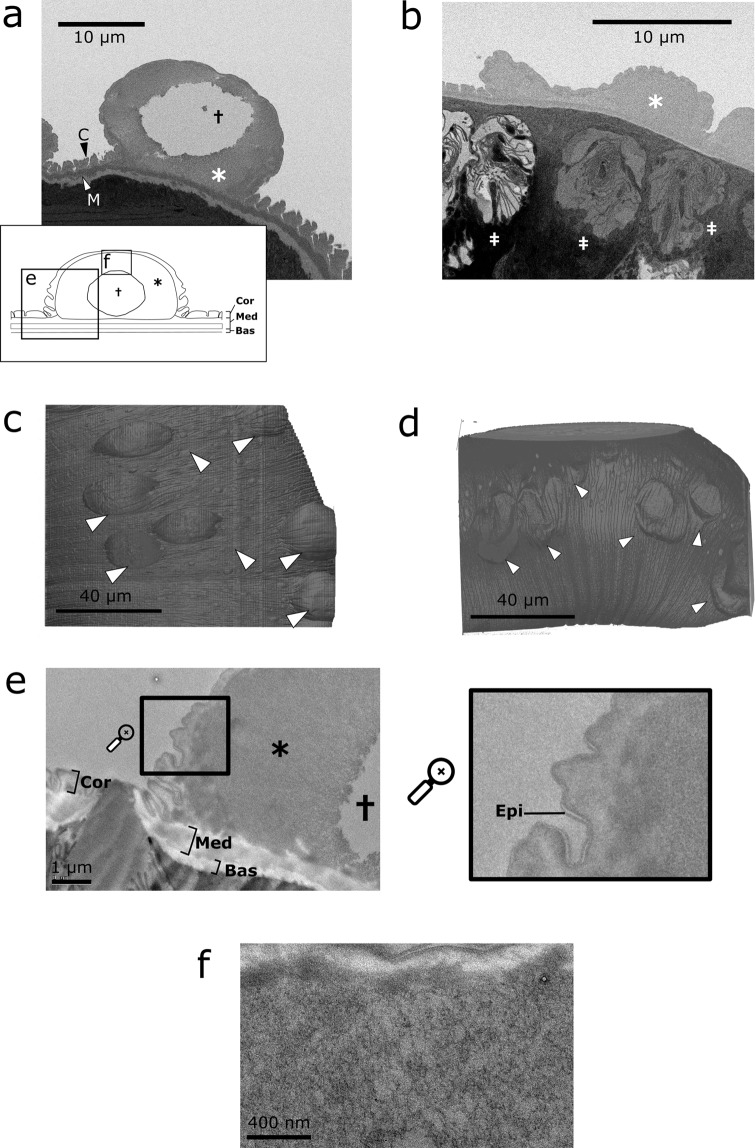
Ultrastructure of cuticular inflations in *T. muris* shown by SBF-SEM and TEM at day 35 PI. (**a**) backscattered electron micrograph of a cuticular inflation presenting its inflated aspect. The inflation develops between the Median (M) and cortical (C) layers of cuticle, and contains both a further layer of cuticular material (*), as well as a void (†). The inset schematic shows the location of the TEM imaging frames shown in (**e**,**f**). (**b**) Backscattered electron micrograph of a cuticular inflation presenting its collapsed aspect. Packing material is present (*), but the void is not. Bacillary cells are present underneath the inflation (‡). (**c**) 3D rendering of the inflated aspect cuticular inflation. (**d**) 3D rendering of the collapsed-aspect inflations. (**e**) TEM micrograph of the cuticular inflation showing the cortical (Cor), basal (Bas), and medial (Med) layers of cuticle. The epicuticle (Epi) is shown in the magnified region. (**f**) the inflation interior layer (*) contains a fine web-like network of fibres.

In the first worm, scanning electron micrographs showed that the outermost cortical layer of cuticle and epicuticle formed an outer layer of the inflation (Fig. 3a,b). Between the cortical and medial layers of cuticle there was a thicker layer of material filling the inflation interior. Within the same worm, inflations in different regions of the bacillary band were imaged, and presented different morphologies. At the anterior of the bacillary band (581 µm from the head), inflations were convex (Fig. 3a,c), and contained voids. However, inflations in a more posterior region of the bacillary band (816 µm from the head) presented a collapsed morphology (Fig. 3d). Like the anterior inflations, these collapsed inflations presented a thick extra layer of cuticle between the median and cortical layers, but did not contain a void (Fig. 3b). The contrast in external morphology seen between the anterior and posterior inflations is highlighted in volume renderings of the entire datasets (Fig. 3c,d). Despite the absence of any Critical point- or Freeze-drying processes, both concave and convex morphologies were preserved in the same worm. Drying was therefore not required for the concave morphology to be exhibited.

At the end of the SBF-SEM acquisition, TEM sections (70 nm) were taken from the block-face and mounted on grids. TEM imaging of these slices revealed the ultrastructure of the inflation at high resolution. Specifically, the structure of the inflation was confirmed as an accumulation of material between the cortical and medial layers of the cuticle. Upon inspection at 11000X magnification (actual image magnification of 15966X), the material filling the inflation was revealed to resemble a web-like network of fibres (Fig. 3e,f). Birefringence of the cuticular inflation interior after histochemical staining with picrosirus red suggested the fibres to be collagen-like (Supplementary Fig. S1). Histochemically this birefringence is understood to be associated with Type-1 collagen fibres; however, there is to date no evidence of Type-1 collagen in nematodes.

**Figure 4:**
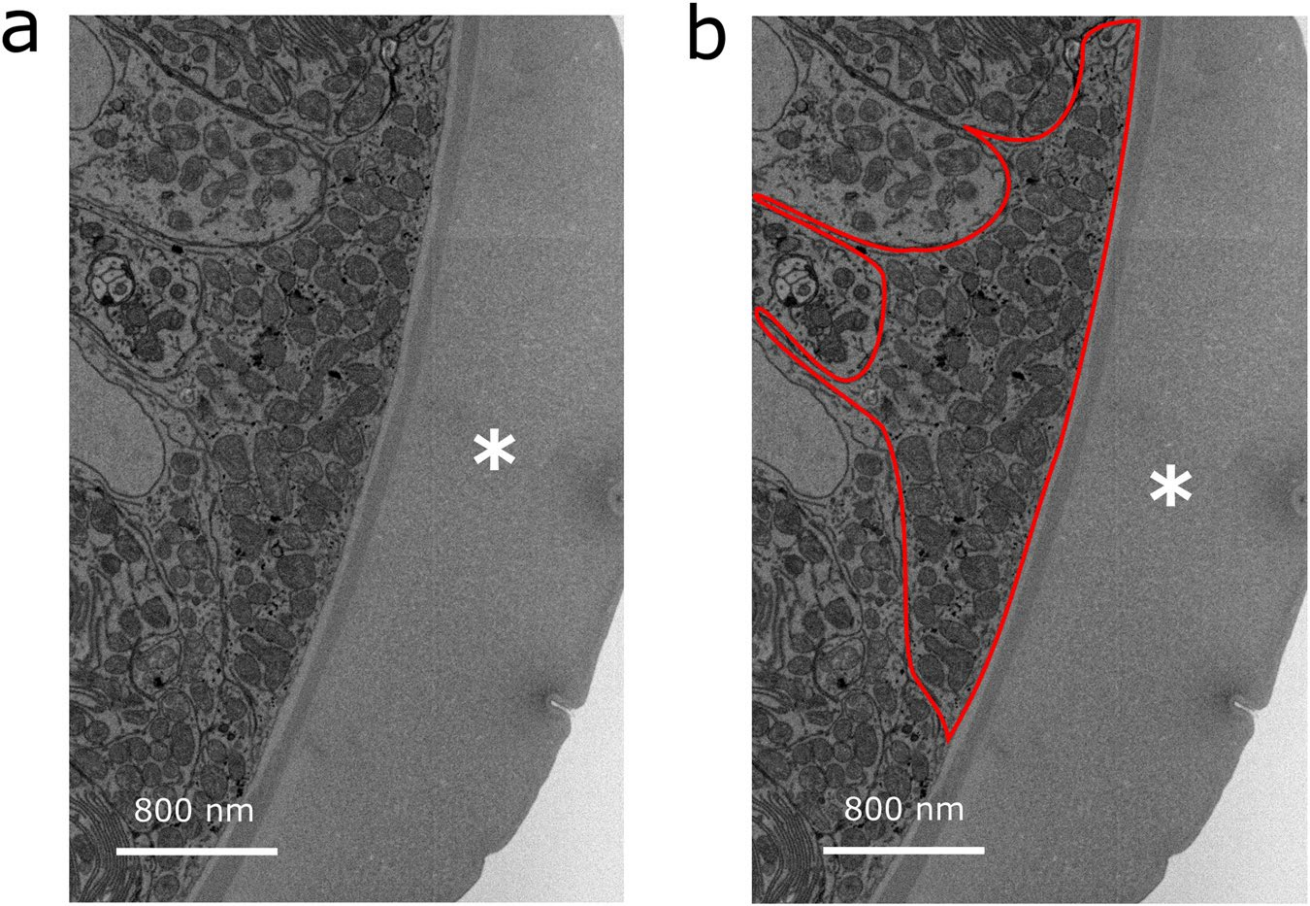
Electron micrograph showing the tissues underlying the cuticular inflation. *T. muris* were extracted at day 35 PI from infected mice and using a correlative imaging workflow, regions of interest were captured by CT-guided SBF-SEM carried out on the cuticular inflations in worms extracted from the host gut. (**a**,**b**) show the ultrastructure beneath the cuticular inflations. In particular, a cell is present with a high number of mitochondria (highlighted in red, (**b**), directly underneath the inflation (*). The cell is not bacillary, and it lacks a folded apical membrane.

Observation of the cell type directly underlying the cuticular inflation was made possible in an investigation of a second day 35 PI worm in which optimal orientation of the inflations was achieved. Directly underlying the cuticular inflations was a cell which, similarly to bacillary cells, exhibited a large quantity of mitochondria (Fig. 4, Supplementary Movie 1), and sparse endoplasmic reticulum (ER). However, this cell also lacked a lamellar zone on its distal surface, which confirmed that it was not a bacillary cell (see bacillary cell in Fig. 1a). The composition of these cells is in contrast to that of the stichocytes, which exhibit notably extensive ER (Supplementary Fig. S2). Similar, mitochondria-rich cells were detected underneath the two other cuticular inflations present in the dataset (Supplementary Fig. S3) These data represent the first 3D electron microscopy dataset of the anterior structures of *Trichuris* spp., and are included in a freely accessible online repository (10.5281/zenodo.3601280).

## Discussion and Conclusions

SEM allowed characterisation of an approximate developmental time-course for the cuticular inflations. Cuticular inflations developed over a period of around 3 days, between days 25 and 28 post-infection. However, the variety of morphologies observed especially in 25 day-old worms suggest that, like moulting, cuticular inflation development may vary in timing by several days between worms^3^. Cuticular inflations do not appear all at once, and the observation of one or two cuticular inflations on worms taken at day 25 post-infection suggests that cuticular inflations may spread along the surface of the worm over the course of several days. Therefore, moulting appears to have little role in cuticular inflation formation, since most inflation development appeared to occur between the second and third moults.

CT-guided SBF-SEM has demonstrated the presence of an additional layer of cuticle between the median and cortical layers. Inspection under the higher magnification of TEM showed that this layer was a loose web-like network of fibres, which picrosirius red histology suggests is collagenous in composition. The morphology of the inflation presents several differences to previously investigated inflations in *T. vulpis* adults^16^. These include the presence of a void in more anterior inflations, and a lack of electron dense granules in the basal region of the inflation. It may be that these features change as the worms mature further beyond the final moult, or alternatively the differences may be species-specific difference. In a single worm, SBF-SEM allowed the identification of both concave and convex cuticular inflations, the structures of which exhibited the absence and presence of a void, respectively. This observation contradicts previous speculation that the collapsed morphology is purely due to the freeze-drying process required to prepare worms for SEM^15^. The consistent sample preparation between the two morphologies of inflation may suggest that both are present on the worm *in vivo*. Alternatively, inflation concavity may be introduced as an artefact in some other part of the staining and embedding process. The extent to which the presence or absence of the fluid filled void is functionally significant remains an open question.

Some have suggested that cuticular inflations act as spacers between the bacillary band and the basal cell membrane of the host^16^, and others have assigned them a glandular role^21,22^. However, cuticular inflation function remains enigmatic. The worm stichosome has extensive ER, indicating its major role in generating secretory products. However, very little ER was observed directly underneath the cuticular inflations. Secreted immunomodulatory proteins are therefore unlikely to originate directly underneath the inflations, and indeed the major secreted protein of *T. muris* seems to exit via the somatic muscles^10^. Notably, cells underneath the inflation exhibited a large number of mitochondria. Therefore, a high capacity for active transport by these cells can be inferred. Since the bacillary cells are the primary site of active glucose uptake by the worm^13^, it is likely that the cells beneath the inflations are engaged in the transport of other species. We may speculate that the worm faces a need to regulate ionic composition of its pseudocoelomic fluid relative to the epithelial cytoplasm in which it lives, in order to maintain appropriate function of tissues such as nerve and muscle. Alternatively, excretion of ammonia in *C. elegans* is mostly accomplished by hypodermal cells^23^, a role which may be applicable to the cells underlying cuticular inflations in *T. muris*. The prospective role of inflations as a site of ion exchange with the intracellular fluid of the host epithelial cells may represent an unexploited opportunity for discovery of novel therapies^24^.

A key question raised by the work is the mechanism for inflation development. Previous authors have remarked upon the similarity of cuticular inflations in *Trichuris* to surface structures in *Gongylonema pulchrum*^16^ and *Syphacia obvelata*^25^, and allude to convergent evolution of the structures. Such comparisons have so far been uninformative of inflation function. Since these studies, the *T. muris* genome has been sequenced^26^, revealing a variety of *T. muris* genes which are involved in different parts of the life-cycle. Comparison of *T. muris* genes with homologs in the highly tractable model nematode *Caenorhabditis elegans* provides a new opportunity to identify and investigate the genetic basis of worm development, including the cuticular inflations. Strains of *C. elegans* which have mutated “blister” genes display cuticular swellings similar to the cuticular inflations seen in *T. muris*. In particular, the genes *bli-5, bli-4* and *dpy-31* have been well-investigated as blister mutants in *C. elegans*^27,28^, and all have homologs in the *T. muris* genome. Understanding spatiotemporal expression of blister gene orthologs in *T. muris* therefore represents a further opportunity for studying inflation development and function, once appropriate genetic tools are developed.

## Methods

### Use of experimental animals

All experiments involving animals were approved by the United Kingdom Home Office and were conducted under the authorities of Home Office personal and project licenses, as per the specifications in the Animals (Scientific Procedures) Act 1986. All methods were performed in accordance with the relevant guidelines and regulations. Severe Combined Immuno-deficient (SCID) mice were bred within the Biological Services Facility (BSF) of the University of Manchester. All animals were kept in specific pathogen free conditions at the University of Manchester BSF, on a 12 hour light/dark cycle. All oral gavage procedures were carried out in the morning between 9:00 and 11:00.

### *Trichuris muris* infection

Eggs of *T. muris* (Edinburgh isolate) were generated by passage, as described in previous literature^29^. To obtain worms for experimental use, Severe Combined Immunodeficient (SCID) mice were administered 200 infective eggs by oral gavage.

### Scanning electron microscopy

Worms were prepared for SEM using an OTO (Osmium-Thiocarbohydrazide-Osmium) protocol designed to impart the maximum structural support to worms throughout the drying process and reduce shrinkage artefacts^30^. The worms were extracted and pooled from two culled hosts at each timepoint 21, 23, 25 and 28 days post-infection and fixed in Karnovsky’s fixative^31^ modified with 0.1 M HEPES for 24 hours. After fixation, worms were washed in ddH_2_O before incubation in 1% OsO_4_ in 0.1 M HEPES. Subsequently samples were washed again before 30 minute incubation in 1% thiocarbohydrazide filtered through a 20 µm pore filter. After washing in ddH_2_O, samples were incubated in 1% aqueous OsO_4_. At this point samples were dehydrated through a graded ethanol series (30%, 50%, 70%, 90%, 100%, 100%, 15 mins each), incubated in 1:1 ethanol: HMDS mixture and then in pure HMDS for 30 mins each. Immediately afterwards, the samples were transferred to fresh HMDS and left to dry within a desiccator. HMDS drying was used due to its comparative ease and comparable results when compared to critical point drying. After drying, the worms were mounted on round SEM stubs and coated with gold-palladium before SEM imaging.

SEM was carried out using an FEI Quanta (Thermo Fisher Scientific, Massachusetts) 250 FEG scanning electron microscope, at 20 kV with a spot size of 3.5 and a chamber pressure of 3.7e^−6^ Torr.

### CT-steered SBF-SEM and TEM

Backscatter and transmission electron microscopy of relevant experimental areas was carried out in a guided manner by using a correlative imaging workflow. In this approach, 3D images obtained by X-ray CT were used to steer sample sectioning as previously described^20^. *T. muris* specimens were prepared using the protocol of Deerinck *et al*.^32^. After 24 hours of fixation in Karnovsky’s fixative modified with 0.1 M HEPES, samples were washed in ddH_2_O before incubation in 1% OsO_4_ and 0.1 M Potassium ferrocyanide in 0.1 M HEPES for 1 hr. Subsequently samples were washed again before 30 minute incubation in 1% Thiocarbohydrazide filtered through a 20 μm pore filter. After washing in ddH_2_O, samples were incubated in 1% aqueous OsO_4_, before again being washed and transferred to a 1% aqueous solution of Uranyl Acetate stored overnight at 4 °C. The samples were washed in ddH_2_O and then incubated in a solution of Walton’s lead aspartate (Walton, 1979) for 1 hr at 60 °C. After dehydration through a graded ethanol series (30%, 50%, 70%, 90%, 100%, 100%, 15 mins each), the samples were incubated in two changes of acetone and then incubated in 25% (Overnight), 50% (Over day), 75% (Overnight), and 100% (6 hr) TAAB 812 Hard resin. Infiltrated samples were then covered in fresh resin in moulds and cured for 24 hours at 60 °C. Excess cured resin surrounding the worm was trimmed down using a hacksaw and razor blades, and the trimmed sample was mounted on a 3View pin.

For correlative imaging, the pin-mounted sample was imaged on the Zeiss Versa 520 X-ray microscope. A 10X optical objective was used and imaging was conducted at an accelerating voltage of 60 kV with 5 W of power. 1601 projections were taken with exposure time of 1.5 seconds each, and the data were reconstructed by filtered back projection. The virtual slices of the tomograms were visualised in Avizo version 9.3 (Thermo Fisher Scientific, Massachusetts) and regions of interest (ROI) were identified. The distance of the ROIs from the tip of the worm head were measured by segmenting the worm using a greyscale threshold, before using the centreline tree module of Avizo to skeletonise the segmented worm (Supplementary Fig. S4). Using CT data as a guide, an ultramicrotome was used to trim to the appropriate position within the tissue block such that the desired region was exposed for the imaging frame. Using a 3view (Gatan, UK) mounted within a Quanta 250 FEG (Thermo Fisher Scientific, Massachusetts) serial images of the blockface were recorded at 3.8 kV, a spot size of 3.5 and a pressure of 0.4 Torr. Pixel size was 12–20 nm and slice thickness was 100 nm. Up to 1000 slices were collected for each data set.

TEM sample preparation was carried out on resin blocks previously imaged by SBF-SEM. Using CT data as a guide, the block-face was trimmed to an appropriate depth to expose a new cuticular inflation and polished with an ultramicrotome. Seventy nanometre slices were cut and without further staining were mounted on grids for TEM imaging. Slices were inspected under a Tecnai 12 Twin Biotwin transmission electron microscope (Thermo Fisher Scientific, Massachusetts), with a Gatan Orius SC1000 camera at 10 µA current 100 kV and 1 second exposure time. Image magnification for TEM ranged from 8000X to 11000X magnification.

### Histology

Composition of cuticular inflations was investigated by histology. Many worms were embedded within one paraffin block in order to increase the chance of capturing inflations. Picrosirius red staining was carried out according to manufacturer’s instructions (Abcam, Cambridge).

## Supplementary information

Supplementary Information.

Supplementary Information2.

## Data Availability

The SBF-SEM dataset of cuticular inflations generated for this study is available in the Zenodo repository (10.5281/zenodo.3601280). Other data from this study is available upon request to the authors.
