# Supplementary material for: Characterisation of cuticular inflation development and ultrastructure in Trichuris muris using correlative X-ray computed tomography and electron microscopy
Source: Sci Rep. 2020 Apr 3;10:5846. doi: 10.1038/s41598-020-61916-0 (PMC7125116; doi:10.1038/s41598-020-61916-0)
Supplement: Supplementary file 1 — Supplementary Information. [file 41598_2020_61916_MOESM1_ESM.docx]

**Supplementary information:**

**Characterisation of cuticular inflation development and composition in *Trichuris muris* using correlative X-ray and electron microscopy**

James D. B. O’Sullivan^1*^, Sheena M. Cruickshank^2^, Toby Starborg^3^, Philip J. Withers^1^, Kathryn J. Else^2*^

1. Photon Science Institute, The University of Manchester, Oxford Road, Manchester, M13 9PY

2. School of Biological Sciences, The University of Manchester, Oxford Road, Manchester, M13 9PT

3. Wellcome Centre for Cell Matrix Research, The University of Manchester, Oxford Road, Manchester, M13 9PT

**Supplementary Figure S1: Picrosirius red staining**

**Figure S1: Picrosirius red staining of paraffin-embedded worms.** Many worms were

embedded within a single paraffin block and sectioned in order to locate cuticular inflations. **a,**

**b)** Cuticular inflations show strong, red picrosirius red staining (arrowheads). **c, d)** Polarised

light microscopy detects the birefringence of collagen stained with picrosirius red. Red

birefringence around and within the inflation shows the position of collagen-like bundles.

However, some inflation contents remained poorly birefringent, despite the positive staining

seen under brightfield illumination.

**Supplementary Figure S2**


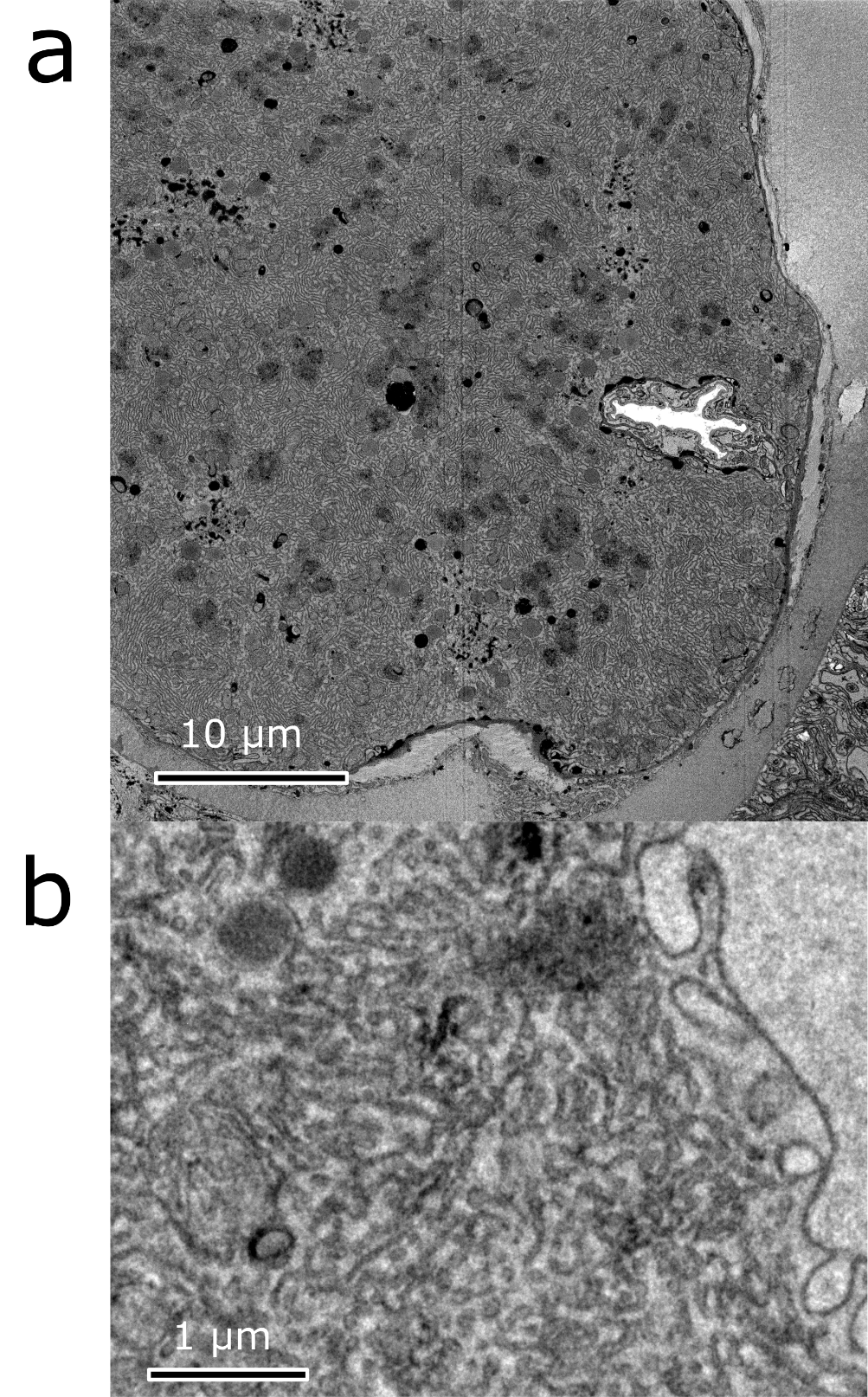


**Figure S2: Extensive rough endoplasmic reticulum in the *T. muris* stichocyte.** A worm was dissected from the caecum of a SCID mouse and prepared en bloc for backscattered scanning electron microscopy and TEM by the protocol of Denk et al. (2003). **a**) By BSEM, the majority of the stichocyte is visible and is packed with membranous structure, likely rough endoplasmic reticulum. **b**) More highly magnified TEM of the stichocyte shows the dense packing of membranes.

**Supplementary Figure S3**

**
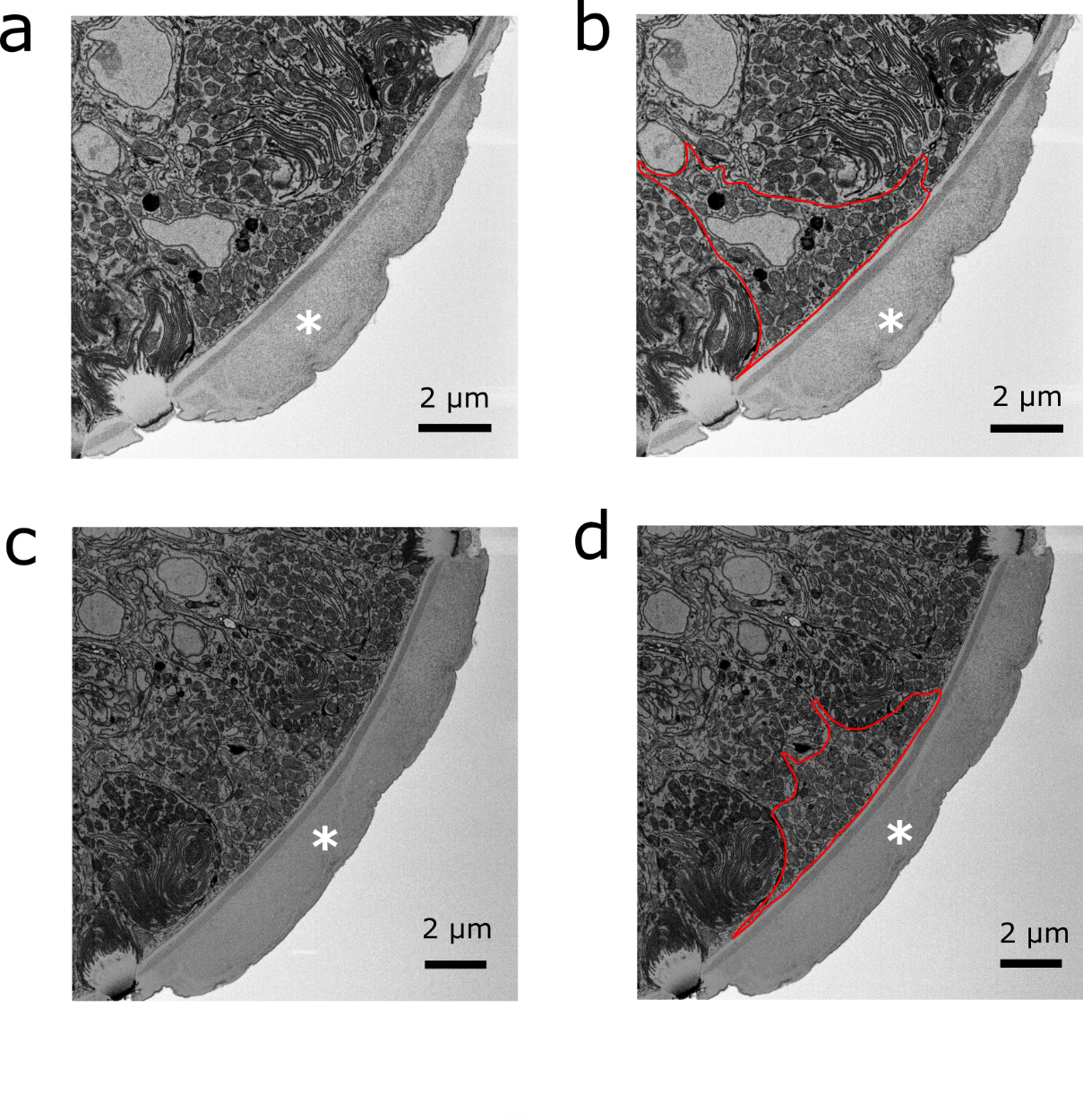
**

**Figure S3: Electron micrograph showing the tissues underlying additional cuticular inflations.** *T. muris* were extracted at day 35 PI from infected mice and using a correlative imaging workflow, regions of interest were captured by CT-guided SBF-SEM carried out on the cuticular inflations in worms extracted from the host gut. **a-d)** show the ultrastructure beneath two cuticular inflations. In particular, a cell is present with a high number of mitochondria (highlighted in red, **b, d**), directly underneath the inflation (*). The cell is not bacillary, and it lacks a folded apical membrane

**Supplementary Figure S4**

**
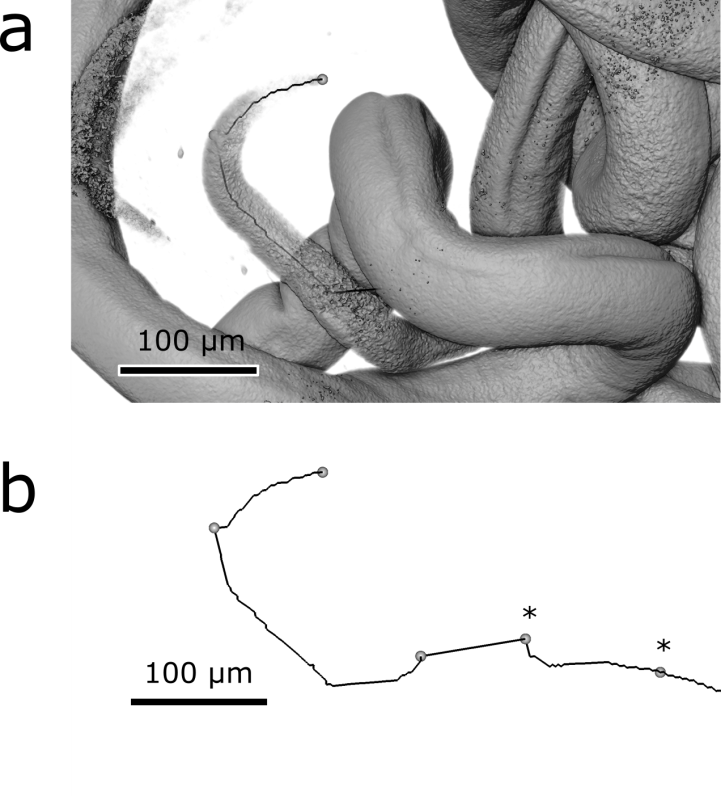
**

**Figure S4: Skeletonisation for quantitative measurements of *T. muris* CT data.** The position of regions of interest (ROIs) on the 35 day PI worm was measured as the distance from the head in a skeletonised model. **a**) The resin-embedded worm (grey) is coiled on itself, making measurement of the ROI locations difficult. **b**) Skeletonisation on the worm allows the distance of the ROIs from the end of the skeleton to be measured. ROI positions are indicated with (*).
